# Long non-coding RNA BCAR4 aggravated proliferation and migration in esophageal squamous cell carcinoma by negatively regulating p53/p21 signaling pathway

**DOI:** 10.1080/21655979.2021.1887645

**Published:** 2021-02-19

**Authors:** Shuo Yan, Jichong Xu, Bingyan Liu, Lin Ma, Hao Feng, Huaqiao Tan, Chun Fang

**Affiliations:** a Department of Interventional Radiology, Tongji Hospital of Tongji University, Shanghai, China; b Department of Interventional Radiology, Tongren Hospital, Shanghai Jiaotong University School of Medicine, Shanghai, China

**Keywords:** LncRNA BCAR4, ESCC, miR-139-3p, ELAVL1, p53/p21 signaling pathway

## Abstract

Long non-coding RNA breast cancer antiestrogen resistance 4 (lncRNA BCAR4) is an independent factor on the survival prognosis of patients with multiple cancers. However, the role of lncRNA BCAR4 in esophageal squamous cell cancer (ESCC) remains unknown. Here, we unraveled that lncRNA BCAR4 was upregulated in ESCC and predicted poor prognosis. Functionally, lncRNA BCAR4 knockdown induced cell apoptosis and G1/S arrest, while inhibited cell proliferation and migration in vitro; conversely, overexpressing lncRNA BCAR4 promoted proliferation and metastasis. Mechanistically, lncRNA BCAR4 sponged miR-139-3p to upregulate ELAVL1, thereby inhibiting p53/p21 pathway in ESCC cells. In conclusion, lncRNA BCAR4 promotes ESCC tumorigenesis via regulating p53/p21 signaling pathway and develops a brand-new biomarker and medicine target for ESCC.

## Graphical Abstract

**Figure:**
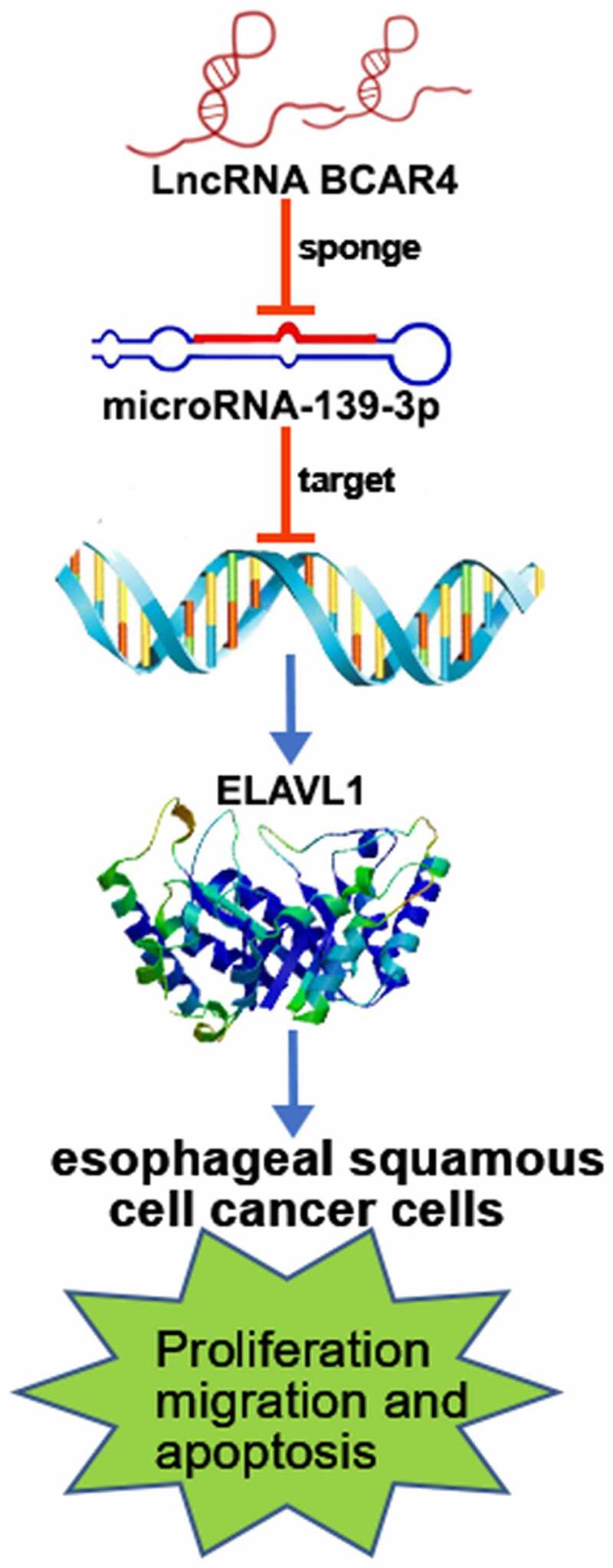

## Introduction

Esophageal carcinoma (EC) ranks eighth in incidence and sixth in mortality of the most common cancer worldwide [1]. It has become clear that EC has the several histological subtypes, includes esophageal adenocarcinoma (EAC), esophageal squamous cell cancer (ESCC), and others [2]. The pathogenesis of EC is still unknown, considering the influence of complex factors, including dietary structure and eating habits, environment, smoking, drinking, genetics, sleep time and so on [3]. Although the application of surgery, radiotherapy, chemotherapy, targeted therapy, and immunotherapy in the comprehensive treatment of cancer is constantly updated, the five-year survival rate of ESCC remains low worldwide, which is about 30–40% [3,4]. Therefore, it is urgent to explore novel biomarkers for diagnosis and prognosis of ESCC.

Long non-coding RNAs (lncRNAs) are non-coding transcripts and play important biological roles in regulating the development of cancer [5–7]. Importantly, increasing studies have reported that lncRNAs is crucial during development and therapeutics of ESCC [8]. For example, over-expressed lncRNA SNHG1 promoted migration and invasion but inhibited apoptosis of ESCC cells [9], and LINC00657 promoted cell proliferation, metastasis, and radio-resistance in ESCC [10]. Notably, lncRNA breast cancer antiestrogen resistance 4 (BCAR4) has been proven to regulate the proliferation, stemness, and metastasis of various types of cancer, including breast cancer (BC), colorectal cancer (CC) and non-small cell lung cancer (NSCLC). The oncogenic role of lncRNA BCAR4 was first confirmed in BC [11–15]. For instance, high BCAR4 mRNA levels predict resistance to tamoxifen therapy and poor outcome in ER*α*-positive BC, reflecting tumor aggressiveness and inducing tumor formation in vivo. Meanwhile, BCAR4 was a strong transforming gene causing estrogen-independent growth and strongly sensitized BC cells to the combination of lapatinib and antiestrogens. Furthermore, BCAR4 drives proliferation of endocrine-resistant BC cells and also play critical roles in the regulation of noncanonical Hedgehog/GLI 2 signal transduction pathways in BC cells. Ouyang *et al* had demonstrated that lncRNA BCAR4 accelerated CC progression via activating Wnt/beta-catenin signaling and maintained the CC cells stemness via miR-665/STAT3 pathway [16,17]. In addition, Li *et al* found that lncRNA BCAR4 promoted the invasion and metastasis of NSCLC via regulating epithelial-mesenchymal transition and Yang *et al* subsequently confirmed that lncRNA BCAR4 increased the progression of NSCLC by targeting glioma-associated oncogene 2 [18,19]. However, no relevant report has been reported the functions of lncRNA BCAR4 on ESCC, and the function of BCAR4 in ESCC is unknown.

MicroRNAs (miRs), ~ 22 nucleotides, are a class of noncoding single-stranded RNA molecules and involved in the regulation of several cancers via binding to lncRNAs [7,20–22]. Moreover, lncRNAs regulate proliferation, metastasis, invasion, and cell death as competing for endogenous RNAs (ceRNAs) in ESCC. For instance, Chu et al [23] report that lncRNA MNX1-AS1 regulated cell malignant progressions by miR-34a/Sirtuin 1 axis and LINC00657 could act as a ceRNA to increase the expression of JunB by binding to miR-615-3p in ESCC [10]. Xia *et al* has been demonstrated that lncRNA TP73-AS1 reducedmiR-139-3p to promote retinoblastoma cell proliferation [24]. In addition, miR-139-3p was predicted to be acted as a tumor suppressor in ESCC [25]. Interestingly, miR-139-3p was the target of BCAR4 in our study through online bio-prediction site. However, the specific role and molecule mechanism of BCAR4 regulating miR-139-3p in ESCC remains unknown.

ELAV Like RNA Binding Protein 1 (ELAVL1), also known as HuR, is highly expressed in several cancers, and can be applied in cancer diagnosis, prognosis, and therapy. Moreover, ELAVL1 has been proven to bind to the 3ʹ-untranslated regions (3ʹUTR) of p53 mRNA and regulate p53 expression [26–29]. However, it is unclear whether ELAVL1 and p53 participated in the progressions of ESCC.

As such, we aim at evaluating the expression of BCAR4 in ESCC tissues, and to explore the functions and molecular mechanisms of BCAR4/miR-139-3p/ELAVL1 axis in ESCC progressions. As a result, lncRNA BCAR4 transcription is enhanced in ESCC tissues and cells. LncRNA BCAR4 RNAi inhibited cell proliferation and migration of ESCC cells. Mechanistically, lncRNA BCAR4 sponging miR-139-3p up-regulated ELAVL1 expression, thereby inhibiting p53/p21 pathway in ESCC cells. Therefore, lncRNA BCAR4 was able to promote malignant progressions *in vivo* and *in vitro* during the development of ESCC.

## Materials and methods

### Bioinformatics analysis based on the cancer genome atlas (TCGA) database and in silico analysis

The mRNA expression data of BCAR4, miR-139-3p and ELAVL1 and the clinical details of patients with ESCA were obtained from StarBase (http://starbase.sysu.edu.cn/index.php). In total, the data of 161 ESCA tissues and 11 normal tissue data were obtained.

DIANA-LncBasev2(http://carolina.imis.athena-innovation.gr/diana_tools/web/index.php?r=lncbasev2%2Findex-predicted) was used to screen the possible miRNA regulators of BCAR4 and DIANA-microT-CDS v5.0 (http://diana.imis.athena-innovation.gr/DianaTools/index.php?r=microT_CDS/index) was used to predict the potential target gene of miR-139-3p.

### Cell culture

Cell lines: human normal esophageal epithelial cells (HEEC) and Human esophageal carcinoma (EC9706, TE-1, and EC109) were purchased from the Chinese Academy of Cell Resource Center (Shanghai, China). All cells were cultured in RPMI-1640 medium (Gibco Life Sciences, Grand Island, NY) added with 10% FBS (Invitrogen, Carlsbad, CA), 100 μg/ml streptomycin, and 100 IU/ml penicillin. All cells were maintained in a humidified incubator containing 5% CO_2_ at 37°C.

In addition, EC109 cells and TE-1 cells were pretreated by 10 mmol/L PFT-α (a p53 inhibitor dissolved in DMSO) for 2 h before transfections and the proliferation and migration were detected.

### Tissue samples collection

Esophageal carcinoma tissues and paired adjacent tissues were obtained from 32 patients at Tongji Hospital of Tongji University. The clinical characteristic information of 32 EC patients are shown in Table S1. The patients did not receive any medical treatment. Samples were frozen in liquid nitrogen immediately after collection, and then stored at −80°C. The study was permitted by the Ethics Committee of Tongji Hospital of Tongji University.

### Plasmid construction and cell transfections

All plasmids used in the present study were synthesized by the GeneChem Company (Shanghai, China) then sub-cloned into pGPH1/Neo (GenePharma, Shanghai, China). The information of three shRNA and shCtrl sequences is listed in Table S2. EC109 or lentivirus vectors infected TE-1 cells with polybrene at a MOI of 20 for 16 h culture and then changed in a new fresh complete growth medium. The infection efficiency was checked by using fluorescence microscope (Olympus), positive cells were used for the subsequent experiments.

miR-139-3p inhibitor (catalog no. 4,464,066; Thermo Fisher Scientific) and miR-139-3p mimic (catalog no. 4,464,084; Thermo Fisher Scientific) were used to accordingly decrease and increase endogenous miR-139-3p levels, respectively.

### Real-time quantitative PCR

Total RNAs were isolated using TRIzol reagent kit (Invitrogen) from tissues and cell lines according to the manufacturer’s instructions. 2.0 μg of total RNA was used to synthesize complementary DNA (cDNA) from using RNA reverse transcription according to the instructions of the PrimeScript^TM^ RT MasterMix kit (Invitrogen). The cDNA samples were used as templates with oligodT primers set to perform the Real-Time PCR amplification using the Applied Biosystems 7500 Sequence Detection system (Thermo Fisher Scientific, Inc.). The final volume of qPCR reaction in each replicate was 10 μL. Relative quantification was achieved by normalization to GAPDH or U6. The relative expression level of target genes was equal to 2^−ΔΔCt^ method. All primers for genes used in our study were synthesized by Sangon. All the sequences of primers were listed in the Table S3. All experiments were repeated triplicates.

### Western blot

The total protein was isolated from cells and tissues in ice-cold RIPA buffer (Beyotime) (150 mM NaCl, 1% NP-40, 0.5% sodium deoxycholate, 0.1% SDS and 50 mM Tris pH 8.0) with inhibitor cocktail (Thermo Fisher Scientific, Inc.), and the protein concentration was determined by the bicinchoninic acid (BCA) Protein Assay kit (Beyotime). 20 μg total protein of each experimental groups was separated by 15% sodium dodecyl sulfate polyacrylamide gel electrophoresis (SDS-PAGE) and subsequently transferred onto PVDF membranes (Millipore). After blocked in 5% skimmed milk overnight, membranes were incubated with primary antibodies at 4°C overnight. The membranes were then incubated with appropriate secondary antibody for 2 h at 37°C after washed three times with 1× TBST. The membranes were visualized using enhanced chemiluminescence (ECL) detection system (Solarbio) and the bands were analyzed using Image J software (NIH). β-actin was used as a loading control. Each experiment was duplicated three times. Antibodies were shown in Table S4.

### MTT assay

Cells (2 × 10^3^ cells/well) were seeded into 96-well plates in 100 µL for 24 h incubation. 20 µL of MTT solution (5 mg/mL, Sigma) was added to cells at 37°C for another 3.5 h incubation. The MTT formazan crystals were dissolved in 200 µL of DMSO. A microplate reader (Tecan) was used to measure the absorbance at 490 nm was under at the following 5 days.

### Cell apoptosis

When the confluence reached 65%~80%, EC109 or TE-1 cells were enriched for cell apoptosis assay. After washed with pre-cooling Annexin V 1× binding buffer (BD Bioscience), cells were centrifuged at 1300 rpm and resuspended in Annexin V 1× binding buffer. Cells were assessed with 5 µL of Annexin V-APC/propidium iodide (PI) double staining reagent (BD Bioscience) for 15 minutes at room temperature and protected from light. A flow cytometer was adopted to distinguish apoptotic cells according to the manufacturer’s instruction. Next, Cell Quest software (version 5.1) is used to analyze data.

### Cell cycle

When the confluence reached 70%~85%, EC109 and TE-1 cells were collected for cell cycle analysis. After washed with pre-cooling PBS, cells were fixed with 70% ethanol at 4°C for 4 h. After washed twice with 4°C ice-cold PBS, cells were resuspended in PBS, seed with the concentration of 1 × 10^6^/ml/well, and then incubated with 800 µL PI solution without light for 1 h at 25°C. Cell cycle distribution was determined using FCM.

### Transwell migration assay

24-well Transwell chambers (Corning, 3422) is used in Migration assay. Infected EC109 (6 × 10^4^ cells/well/100 µL) or TE-1(8 × 10^4^ cells/well/100 µL) cells were seeded on the chambers at upper-side with serum-free RPMI 1640 medium. The chambers at lower-side were filled with 600 µL RPMI 1640 with 30% FBS medium. After 24 h, cells on the upper side of the filter were removed, and cells on the lower-side chamber were fixed with 1% paraformaldehyde for 1 h, washed five times with PBS and further stained with crystal violet for 40 min. The number of stained cells was counted in three randomly individual fields under a light microscope. We further analyzed the images of taken cells.

### Xenograft model

EC109 cells transfected with shBCAR4-1 or shCtrl were washed, and resuspended in saline. Ethics Committee of Tongji Hospital of Tongji University permitted all animal procedures the. Female BALB/c nude mice xenograft model (5–6 weeks old) were formulated by subcutaneous injection of 0.2 mL exponentially growing lentivirus infected EC109 cell suspensions at a destiny of 1 × 10^7^ cell/mL. The mice were observed every day and tumor growth data were measured twice weekly after 16 days of injection using a caliper. Tumor photographing tumor volumes and weighting were conducted on day 16 post-inoculation immediately after euthanizing tumor-bearing mice. Tumor volumes (V) were estimated as the formula: ½ (Length × Width^2^). Part of the tumor tissues were sed for Ki-67 immunostaining, and other part of tumor tissues were cut and frozen immediately for RNA extraction to assess BCAR4 expression.

### Ki-67 staining assay

3% PBS-H_2_O_2_ is used to block Tissue slides. First antibody of Ki-67 is incubated with slides at 4°C overnight. HRP goat anti-rabbit IgG were then incubated with slides at 37°C for 2 h. Finally, slides were finally stained with Hematoxylin (# BA4041, Baso) and Eosin (# BA4022, Baso).

### Dual luciferase reporter assays

Luciferase (Promega) assay was performed according to following the manufacturer’s instructions. Cells (1 × 10^6^ cells/well) were planted into 24-well plates and then transfected with each individual vector (pGL3-basic, Promega) containing a wild-type (wt)-BCAR4 or a mutant (mut)-BCAR4. Luciferase reporter gene vectors (pRL-TK, Promega) containing a wildtype-ELAVL1 3ʹ-UTR or a mutant-ELAVL1 3ʹ-UTR were transfected into EC109 cells using Lipofectamine® 2000. Aliquots of 50 nM of miR-139-3p mimic or NC was co-transfected with reporter plasmids. After 48 h incubation, the luciferase fluorescence abundance of reporter plasmids was recorded. The relative luciferase fluorescence abundance was normalized by Renilla luciferase fluorescence abundance.

### Human apoptosis antibody array analysis

We knockdown BCAR4 expression in ESCC cell line EC109 by shRNA. After lysis of cell lysates, protein concentration was analyzed with the BCA protein assay kit (Beyotime). The proteins related to apoptosis signaling pathways of lncRNA BCAR4 in EC109 cells were surveyed by apoptosis kit (ab134001, Abcam, Cambridge, MA) with to the manufacturer’s protocols. The array target signaling pathway map involved can be retrieved at the manufacturer’s homepage (https://www.abcam.cn/human-apoptosis-antibody-array-membrane-43-targets-ab134001.html). The ImageJ is used to analyze the intensity score of each individual array spot.

### Statistical analysis

Data were expressed as mean ± standard deviation with at least three independent experiments. SPSS 22.0 (IBM, SPSS) and GraphPad Prism 7 (GraphPad Software) were used to analyze data. Student’s t-test and one-way ANOVA were used to compare data according to different experiment set. *P* < 0.05 was considered to be significantly different.

## Results

### BCAR4 is highly expressed in ESCC tissues and cells

**Figure 1.**
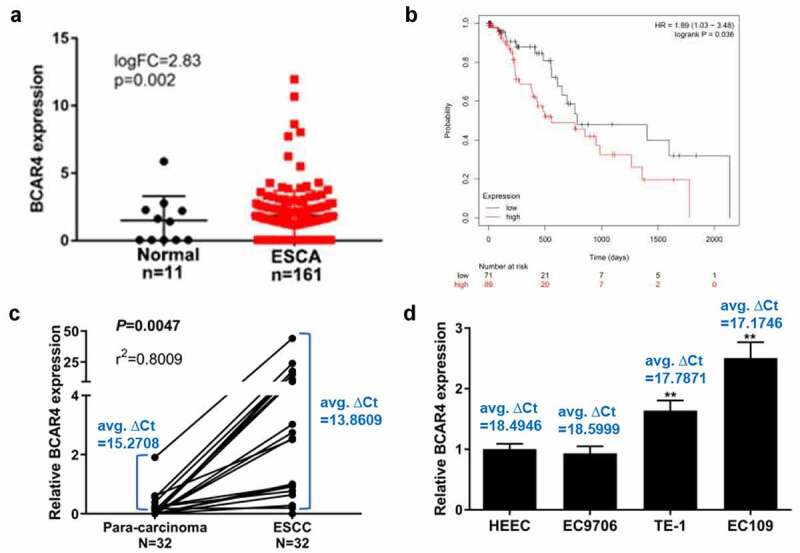
BCAR4 was highly expressed in ESCC tissues and cells. (a) The expression level of BCAR4 in clinical ESCA (Esophageal Carcinoma) tissues compared with the normal tissues. Expression data of BCAR4 were downloaded from the Cancer Genome Atlas (TCGA). (b) Kaplan–Meier analysis was used to determine the OS curve of patients with high BCAR4-expressing ESCC tumors vs. low BCAR4-expressing tumors with the data from TCGA. Statistical analysis was performed by log-rank test in a SPSS 22 for Windows. (c)The relative expression of BCAR4 in 32 paired ESCC tissues and adjacent para-carcinoma tissues obtained from ESCC patients was checked by RT-qPCR. (d) Relative expression of BCAR4 was detected in three ESCC cell lines (EC9706, TE-1 and EC109) and the normal human esophageal epithelial cell line HECC by RT-qPCR. n = 3, **p < 0.01

RT-qPCR, quantitative real‐time polymerase chain reaction; OS, overall survival; ESCC, esophageal squamous cell cancer.

To explore the roles of BCAR4 in ESCC, we first analyzed the expression patterns of BCAR4 in ESCC tissue samples with relative bioinformatics methods based on the TCGA data. The expression level of BCAR4 was obviously increased in clinical ESCC tissues compared with the non-tumor tissues (Figure 1a). The overall survival curve determined by Kaplan-Meier method and log-rank test uncovered that a poorer outcome was observed in the patients with higher BCAR4 expression (Figure 1b). Furthermore, the mRNA level of BCAR4 in 32 paired of tumor and tumor-adjacent tissues isolated from ESCC patients were recorded, and found that BCAR4 was visibly enhanced in ESCC tissues when compared with the adjacent tissues (Figure 1c). Moreover, BCAR4 was also overexpressed in three ESCC cell lines, includes EC9706, TE-1 and EC109, in comparison to the HECC cells (Figure 1d). These data implied that BCAR4 is significantly overexpressed in tissues ESCC tissues and cell lines and may be crucial for ESCC development.

### BCAR4 promotes ESCC cell proliferation and migration

**Figure 2.**
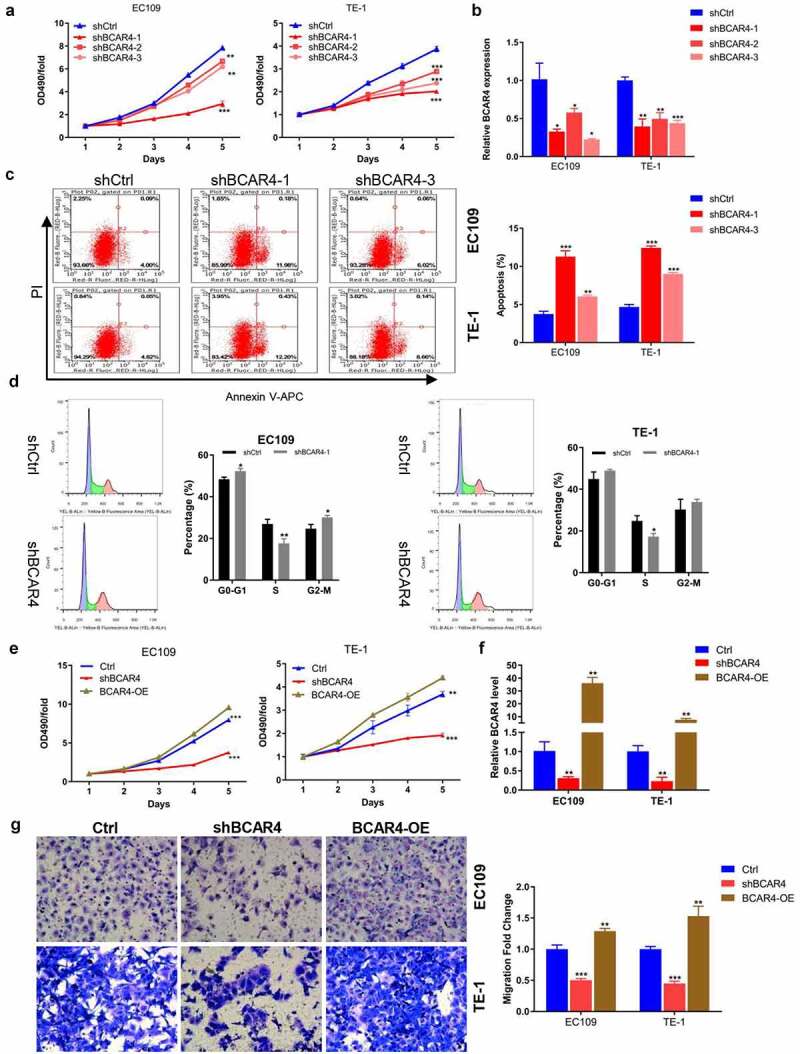
BCAR4 promotes ESCC cell proliferation and migration. (a) The viability of BCAR4 knockdown EC109 and TE-1 cells was detected by MTT assay, compared with the control(shCtrl) group. n = 3, **p < 0.01, ***p < 0.001. (b) Relative expression of BCAR4 was assayed in EC109 and TE-1 cells infected with shBCAR4 or shCtrl by RT-qPCR. n = 3, *p < 0.05, **p < 0.01, ***p < 0.001. (c) Cell apoptosis rates in BCAR4 silenced EC109 and TE-1 cells were analyzed by flow cytometry analysis, compared with the shCtrl group. n = 3, **p < 0.01, ***p < 0.01. (d) Cell cycle in BCAR4 silenced EC109 and TE-1 cells were analyzed by flow cytometry analysis, compared with the shCtrl group. n = 3, *p < 0.05, **p < 0.01. (e) The viability of EC109 and TE-1 cells was stronger in the BCAR4-OE group but weaker in the shBCAR4 group, compared with the Ctrl (GFP-vector) group. n = 3, **p < 0.01, ***p < 0.001. (f) The PCR results showed that ESCC cell lines were successfully infected with BCAR4-OE lentivirus or shBCAR4 lentivirus, compared with the Ctrl group. n = 3, **p < 0.01. (g) The migration cells were obviously more in the BCAR4-OE group, while the migration cells were less in the shBCAR4 group. n = 3, **p < 0.01, ***p < 0.001, compared with the Ctrl group

To further explore the biological functions of BCAR4 in ESCC, we constructed three shRNAs targeting BCAR4 (shBCAR4-1, −2, and −3) and BCAR4 overexpressing plasmids (BCAR4-OE). We first verified that shBCAR4-1 and shBCAR4-3 were the more effective interference targets than shBCAR4-2 by the detection of MTT assay and RT-qPCR (Figure 2a and b). The following cell apoptosis assay by flow cytometry analysis confirmed that BCAR4 knockdown induced cell apoptosis in EC109 and TE-1 cells and further verified shBCAR4-1 was the most effective interference target comparing to shBCAR4-3 (Figure 2c). Therefore, shBCAR4-1 was selected for the follow-up experiments. In addition, we found that the percent of cells numbers in S phase of the shBCAR4 group was distinctly decreased (Figure 2d). Furthermore, the MTT results proved that the viability of EC109 and TE-1 cells was significantly increased in the BCAR4-OE group but reduced in the shBCAR4 group, when compared with the Ctrl group (Figure 2e). The RT-qPCR results suggested that EC109 and TE-1 cells were successfully infected by BCAR4-OE lentivirus or shBCAR4 lentivirus (figure 2f). Moreover, in both two ESCC cell lines, more migration cells were observed in the BCAR4-OE group, while the less migration cells were showed in the shBCAR4 group, when compared to the Ctrl group (Figure 2g). These findings indicated that BCAR4 participated in malignant progressions in ESCC development.

### Knockdown of BCAR4 suppressed tumor growth in vivo

**Figure 3.**
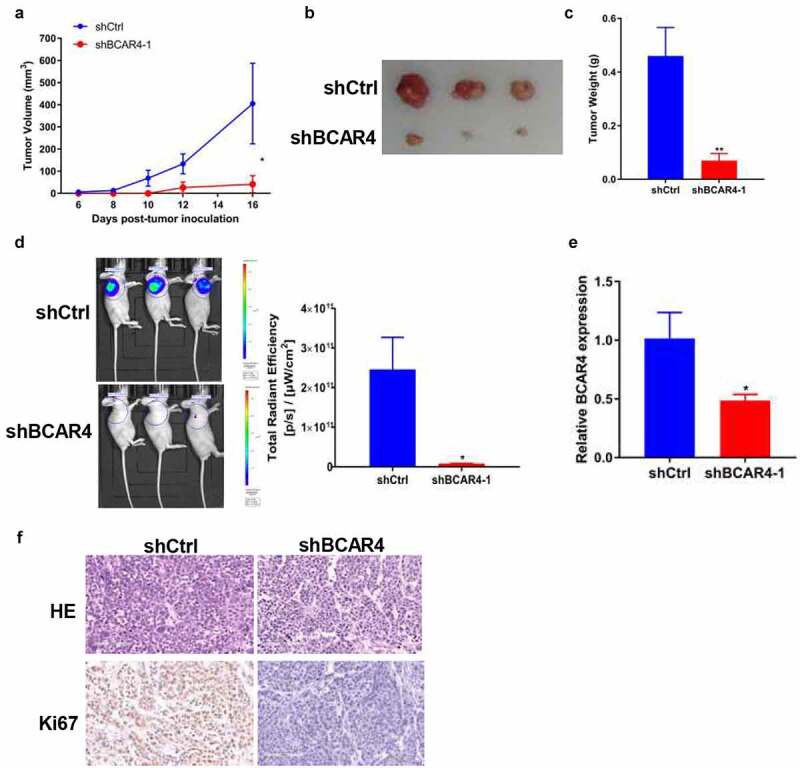
Knockdown of BCAR4 suppressed tumor growth and metastasis in vivo. BCAR4 knockdown EC109 cells or control cells were subcutaneously injected into nude mice to form solid tumors (*n* = 3 for each group). (a) Tumor volumes and (b) tumor photographs were obtained, and (c) tumor weights were measured in shBCAR4 and shCtrl groups. *p < 0.05, **p < 0.01, compared with the shCtrl group. (d) On the last day of analysis, the representative images of in vivo luciferase-labeled cells imaging were photo and the total radiant efficiency of tumor were analyzed. *p < 0.05, compared with the shCtrl group. (e) Levels of BCAR4 in tumor tissues dissected from nude mice treated with shBCAR4 or shCtrl were detected using RT-qPCR. *p < 0.05, compared with the shCtrl group. (f) HE staining was used to observe histomorphology in tumor tissues of mice treated with BCAR4 knockdown EC109 cells or control vector cells. Immunohistochemical staining of Ki67 in tumor tissues dissected from nude mice treated with BCAR4 knockdown EC109 cells or control vector cells

To evaluate the effects of BCAR4 on tumor growth *in vivo*, EC109 cells stable expressed with shBCAR4 or shCtrl was injected into nude mice subcutaneously to form solid tumors. On day 6 after implantation, we recorded the tumor lengths and widths every 2 days to obtain 6. The tumor growth curve showed significant reduction in the BCAR4 knockdown group compared with shCtrl group (Figure 3a). Subsequently, the tumors were isolated and the sizes are recorded in Figure 3b and weights were evaluated. As shown in Figure 3c, the tumor weight in the shBCAR4 group was significantly lower than those of the tumors in the shCtrl group. Meanwhile, the fluorescence intensity of *in vivo* imaging and the total radiant efficiency also reflected the mean volume and mass of tumors, which was decreased by BCAR4 knockdown (Figure 3d). Efficient knockdown of BCAR4 in EC109 cells following shRNA interference action was verified by qRT-PCR in tumor tissues (Figure 3e). The proliferative abilities of the tumor cells were measured by immunohistochemical staining of Ki-67 in isolated tumors. Results showed that the staining intensities were reduced in tumors from the shBCAR4 group when compared with that in the shCtrl group (figure 3f). Thus, BCAR4 plays important roles in ESCC growth *in vivo* and indicates that BCAR4 may promote in ESCC progression.

### BCAR4 modulates miR-139-3p/ELAVL1 axis in ESCC cells

**Figure 4.**
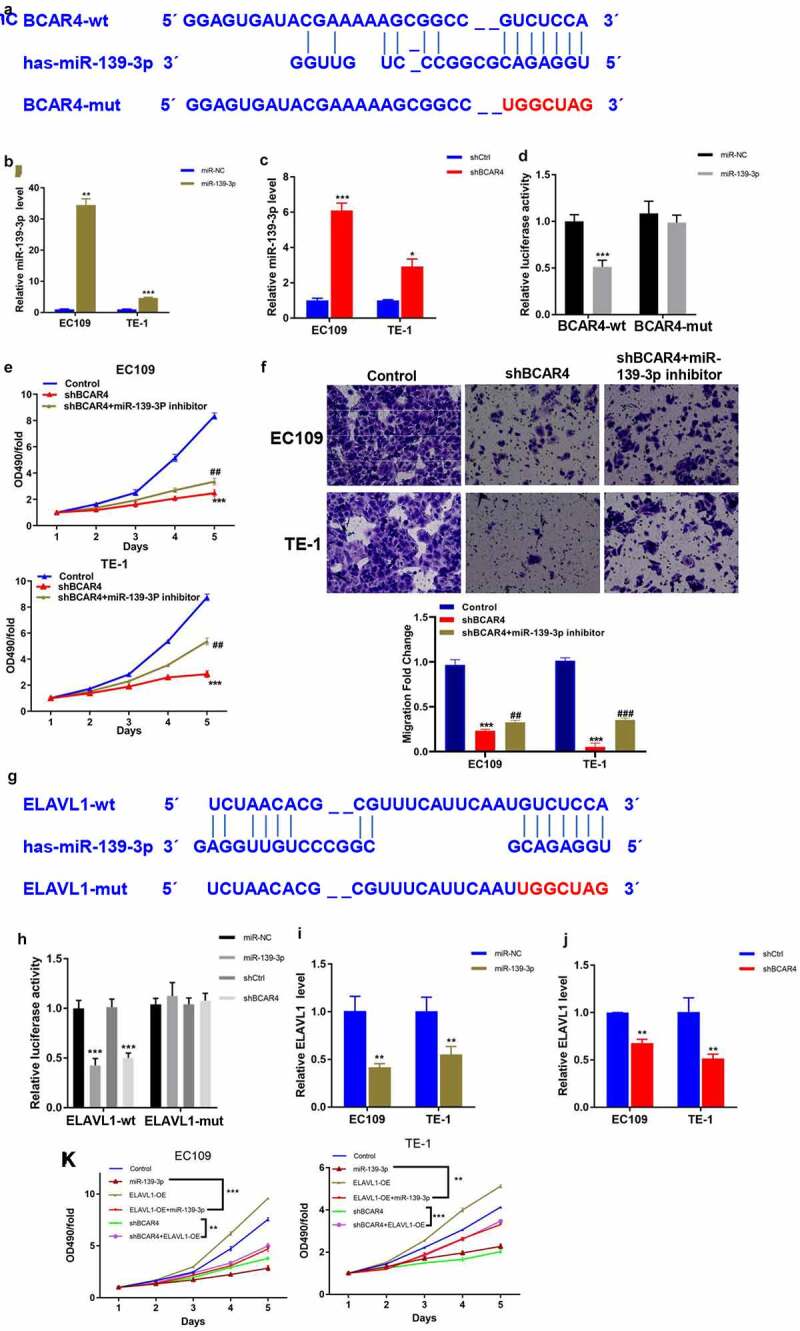
BCAR4 modulates miR-139-3p/ELAVL1 pathway in ESCC cells. (a). BCAR4 wide-type (BCAR4-wt) and the mutated-type (BCAR4-mut) sequences in the has-miR-139-3p binding sites were shown according to DIANA-LncBase v2. (b). Relative expression of miR-139-3p was examined using RT-PCR assay after miR-139-3p overexpression. n = 3, **p < 0.01, ***p < 0.001, compared with the miR-NC group. (c). BCAR4 knockdown increased the expression of miR-139-3p in both EC109 and TE-1 cells. n = 3, *p < 0.05, ***p < 0.001, compared with the shCtrl group. (d). The dual luciferase reporter assay showed that overexpression of miR-139-3p repressed relative luciferase activity of EC109 cells transfected with BCAR4-wt or BCAR4-mut. n = 6, ***p < 0.001, compared with the miR-NC group. (e). The viability of EC109 and TE-1 cells in BCAR4 knockdown or BCAR4 plus miR-139-3p knockdown groups was detected by MTT assay. n = 3, ***p < 0.001, compared with the Ctrl group; ^##^p < 0.01, compared with the shBCAR4 group. (f). The migration cells of EC109 and TE-1 cells in BCAR4 knockdown or BCAR4 plus miR-139-3p knockdown groups was detected by Transwell assay. n = 3, ***p < 0.001, compared with the Ctrl group; ^##^p < 0.01, ^###^p < 0.001, compared with the shBCAR4 group. (g). ELAVL1 wide-type (ELAVL1-wt) and the mutated-type (ELAVL1-mut) sequences in the has-miR-139-3p binding sites were shown according to DIANA-microT-CDS. (h). The dual luciferase reporter assay showed that overexpression of miR-139-3p or BCAR4 knockdown repressed relative luciferase activity of EC109 cells transfected with ELAVL1-wt. n = 6, ***p < 0.001, compared with the miR-NC group or shCtrl. (i). miR-139-3p overexpression reduced the expression of ELAVL1 in both EC109 and TE-1 cells. n = 3, **p < 0.01, compared with the miR-NC group. (j). BCAR4 knockdown reduced the expression of ELAVL1 in both EC109 and TE-1 cells. n = 3, **p < 0.01, compared with the shCtrl group. (k). MTT assay was using to detect the cell viability of ESCC cells after transfecting indicated plasmids. n = 3, **p < 0.01, ***p < 0.001

To explore the underlying mechanisms of BCAR4 in the ESCC progression, the DIANA-LncBase v2 was used to screen the possible miRNA regulators of BCAR4 and we found that miR-139-3p was predicted to have numerous probabilities of binding to BCAR4 (Figure 4a). The results of luciferase assays demonstrated that enhanced expression of miR-139-3p led to reduction of luciferase fluorescence intensity in the BCAR4-wt group, while it did not affect the activity in BCAR4-mut group (Figure 4d). Furthermore, an overexpression plasmid of miR-139-3p was used to assess the relationship between BCAR4 and miR-139-3p and data showed miR-139-3p mimic could significantly elevate miR-139-3p expression both in EC109 and TE-1 cells (Figure 4b). Besides, knockdown of BCAR4 increased miR-139-3p expression (Figure 4c). Then, a rescue experiment was carried out. As shown in Figure 4e and f, we observed that knockdown of miR-139-3p could reverse the inhibitory effect of shBCAR4 on ESCC cell proliferation and migration, and then restore cell vitality. These findings confirmed that BCAR4 directly modulated the expression of miR-139-3p in ESCC cells.

**Figure 5.**
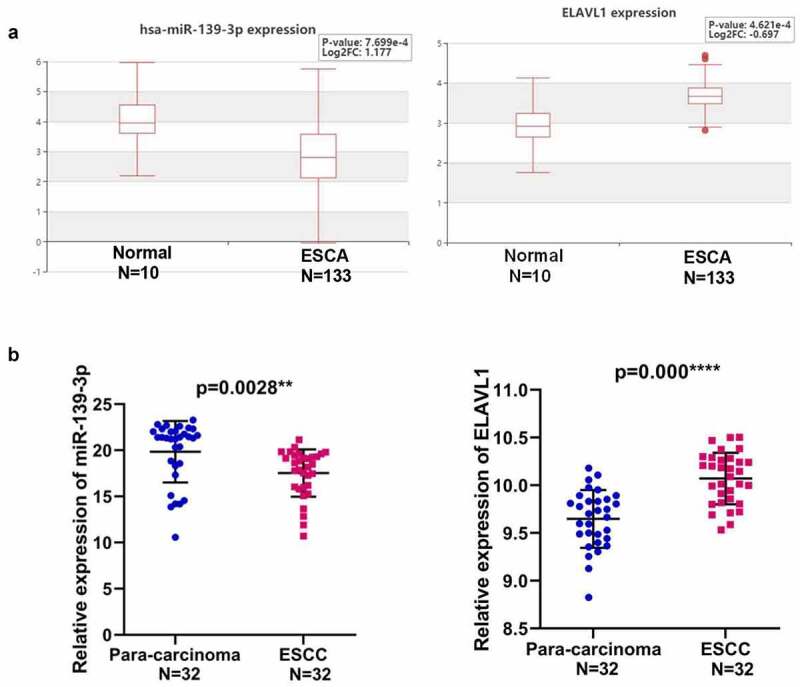
Expression of miR-139-3p and ELAVL1 in ESCC. (a). The expression level of has-miR-139-3p and ELAVL1 in the ESCA (Esophageal Carcinoma) tissues compared with the normal tissues using HCMDB datasets. (b). The relative expression of miR-139-3p and ELAVL1 in 32 paired ESCC tissues and adjacent para-carcinoma tissues obtained from ESCC patients was checked by RT-qPCR. **p < 0.01, ***p < 0.01. ESCC, esophageal squamous cell cancer

To identify the potential target gene of miR-139-3p underlying BCAR4, we used DIANA-microT-CDS to predict the target gene of miR-139-3p and found ELAVL1 was a potential downstream gene of miR-139-3p (Figure 4g). Luciferase assays and RT-qPCR results confirmed that miR-139-3p overexpression or BCAR4 knockdown remarkably reduced the luciferase activity of wild-type ELAVL1 and the mRNA expression of ELAVL1 (Figure 4h–j). Consistently, overexpression of miR-139-3p or knockdown of BCAR4 overtly reduced the cell viability of ESCC cells, while overexpression of ELAVL1 recovered the decreased cell viability induced by miR-139-3p overexpression or shBCAR4 both in EC109 and TE-1 cells (Figure 4k).Expectedly, miR-139-3p was downregulated in ESCA tissues while ELAVL1 was overexpressed in Esophageal Carcinoma (ESCA) from the HCMDB datasets (Figure 5a). The same trend was observed in 32 pairs of clinical samples (Figure 5b). Collectively, data in the present study indicated that ELAVL1 may be downstream gene of miR-139-3p sponging to BCAR4, which are involved in tumor progressions in ESCC.

### BCAR4 promotes ESCC via regulating p53/p21 signaling pathway

**Figure 6.**
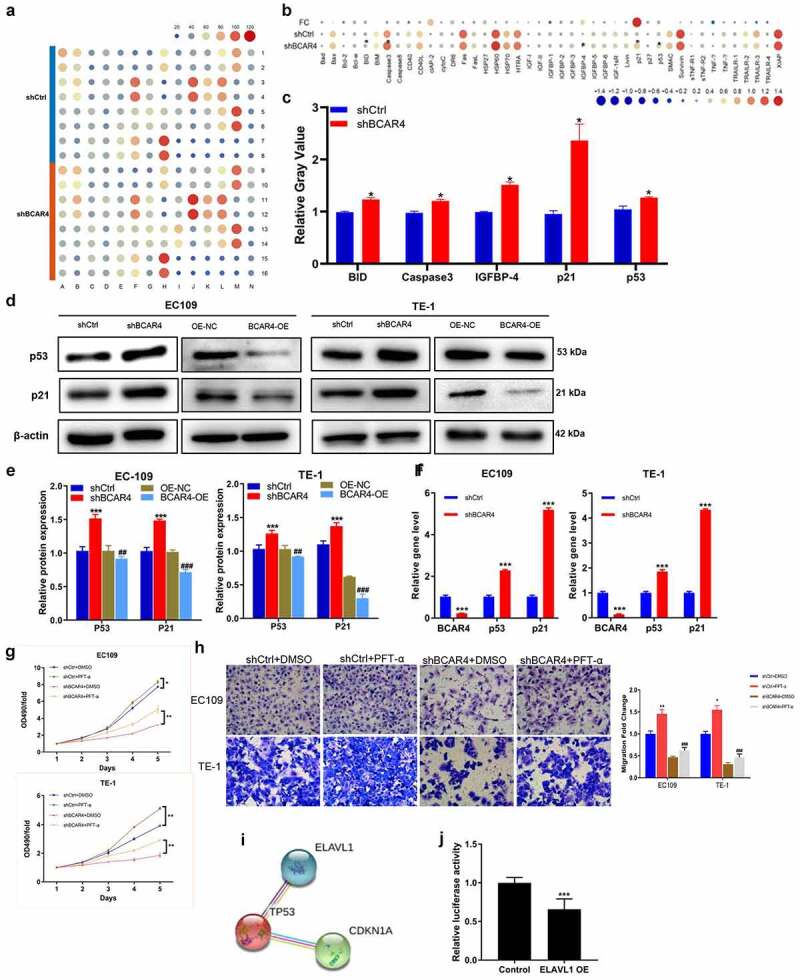
Exploring the downstream regulatory mechanism of BCAR4. (a) Human apoptosis antibody array was performed to analyze the differential expression of 43 human apoptotic markers in EC109 cells between shBCAR4 and shCtrl groups. (b) The relative protein levels of 43 human apoptotic markers in EC109 cells between shBCAR4 and shCtrl groups. FC, fold change. (c) 5 differential expression proteins in EC109 cells between shBCAR4 and shCtrl groups. (|FC|>20%, p < 0.05). (d) Western Blotting results showed protein expression in EC109 and TE-1 cells, infected with shBCAR4 or shCtrl. (e) Intensities of protein bands standardized to those of β-actin and expressed as relative band intensities. n = 3, *p < 0.05, **p < 0.01, ***p < 0.01. (f) The PCR results showed that indicated gene expression in EC109 and TE-1 cells, infected with shBCAR4 or shCtrl. n = 3, ***p < 0.01. (g) Growth curve analysis showed cell growth of EC109 and TE-1 cells infected with shBCAR4 or shCtrl, and treated with PFT-a or not. Cells were pretreated by 10 mmol/L PFT-α (a p53 inhibitor dissolved in DMSO) for 2 hours. n = 3, *p < 0.05, **p < 0.01. (h) Transwell assays showed cell migration of EC109 and TE-1 cells infected with shBCAR4 or shCtrl, and treated with PFT-a or not. Cells were pretreated by 10 mmol/L PFT-α (a p53 inhibitor dissolved in DMSO) for 2 hours. n = 3, *p < 0.05, **p < 0.01, compared with shCtrl+ DMSO groups; ^###^*p* < 0.001, compared with shBCAR4+ DMSO groups. (i) Using the STRING program to analyze potential interaction between ELAVL1 and the p53 (also known as TP53). (j) A dual luciferase assay was performed to determine the effect of ELAVL1 overexpression on transcriptional activity of TP53 in EC109 cells

To explore the underlying mechanism of the regulation ability of BCAR4 silence in ESCC, the contrasting expression pattern of 43 human apoptotic-related proteins in EC109 cells was investigated using Human apoptosis antibody array. As shown in Figure 6a–c, the pro-apoptotic proteins were overtly increased by BCAR4 knockdown, includes BID, Caspase-3, IGFBP-4, p21, and p53; among them, p21 was strongly upregulated in EC109 cells. Western blotting and RT-PCR results revealed that BCAR4 knockdown increased the protein and mRNA level of p53 and p21 in both EC109 and TE-1 cells (Figure 6d–f). Meanwhile, the inhibitory ability of BCAR4 knockdown on cell viability and migration were attenuated by the p53 inhibitory PFT-α (Figure 6g and h). Importantly, ELAVL1 appeared to be a potential upstream regulatory factor for p53 through the String program (Figure 6i). In addition, upon ELAVL1 overexpression, the luciferase fluorescence intensity in the p53-driven luciferase assay was remarkably decreased (Figure 6j). All these findings highlighted p53/p21 signaling as a potential downstream pathway of EVAVL1, which are regulated by BCAR4/miR-139-3p participating in cell apoptosis in ESCC progression.

## Discussion

Bioinformatics analysis basing on public TCGA databases indicates that BCAR4 was highly expressed in ESCC tissues and closely correlated with poor prognosis of ESCC patients, suggesting that BCAR4 may serve as an oncogene to promote ESCC malignant progression. Furthermore, BCAR4 knockdown- and overexpressed – ESCC cell models were established to confirm the functions of BCAR4 during ESCC development. It was shown that BCAR4 knockdown induced cell apoptosis and arrest cells at the G1-S phases, whereas inhibited cell viability and migration. Conversely, BCAR4 overexpression promoted cell viability and migration. Importantly, knockdown of miR-139-3p could reverse the inhibitory effect of shBCAR4 on ESCC cell proliferation and migration. Mechanistically, BCAR4 participates in tumor progression via sponging miR-139-3p to upregulate ELAVL1 in ESCC. In addition, BCAR4 and ELAVL1 were involved in activating p53/p21 signaling pathway. Collectively, lncRNA BCAR4 promotes ESCC tumorigenesis via regulating p53/p21 signaling pathway and plays as a new prognostic biomarker therapeutic target for ESCC.

LncRNAs are known to function as ceRNAs to regulate downstream genes (miRNAs or mRNAs) expression and then influenced the biological functions of several carcinomas. Accumulating evidence has revealed the oncogenic role of BCAR4 in various studies. For instance, Zhang et al [30] hold that the BCAR4/miR-370-3p/Wnt7a pathway is crucial in regulating the proliferation of bladder cancer cells regarding the oncogenic lncRNA. Moreover, Wei et al [31] found that BCAR4 promoted the progression via activating EGFR/PI3K/AKT pathway, which indicated that BCAR4 could be an effective new target for glioma patients' treatment. In our study, BCAR4 was proved to bind miR-139-3p thereby targeting ELAVL1, which was consistent with a previous research that miR-139-3p inhibited cancer progress by reducing ELAVL1 expression [32]. Consistently, overexpression of miR-139-3p or knockdown of BCAR4 overtly reduced cell viability of ESCC cells, which were rescued by overexpression of ELAVL1 by reducing cell viability. Therefore, the BCAR4/miR-139-3p/ELAVL1 axis may serve as crucial role in promoting ESCC.

Escape from apoptosis is an inducible factor for the occurrence of cancer [33]. This study also demonstrated that BCAR4 RNAi could upregulate the protein expression of human pro-apoptotic markers, includes BID, Caspase-3, IGFBP-4, p21, and p53. It was well known that BID-encoded death agonist could heterodimerize with either apoptosis-agonist BAX or -antagonist BCL2, thereby directly triggering apoptosis [34]. Caspase-3 was considered as a vital executioner of apoptosis which was activated in apoptotic cells after being stimulated by several signals and pathways [35]. IGFBP4 is the smallest and a unique member among the human insulin-like growth factor binding proteins (IGFBPs), which can also act independently away from IGFs and modulate cell proliferation, survival and migration, and so on [36]. p21, a CDK inhibitor, participated in cell cycle progression and could play a crucial role in tumor development by p53 pathways [37–41]. To sum up, cell apoptosis induced by BCAR4 knockdown is a complex process and involved with multiple apoptotic proteins. Here, p21 was one of the most strongly upregulated genes and p53 was upregulated at the same time, we focused on the role of p53/p21 signaling in ESCC underlying BCAR4/miR-139-3p/ELAVL1 axis. We confirmed that BCAR4 knockdown can increase the transcripts abundance of p53 and p21 in these two ESCC cell lines. Furthermore, the inhibitory ability of BCAR4 RNAi on ESCC cells were attenuated by the p53 inhibitory PFT-α. Notably, combining the results of string program and luciferase reporter assay, we confirmed that ELAVL1 directly bind to p53 thereby participating in ESCC progression. All in all, BCAR4 promotes cell proliferation and migration via regulating miR-139-3p/ELAVL1 axis and p53/p21 signaling pathway in ESCC.

## Conclusion

In summary, BCAR4 may act as a potential biomarker for diagnosis and prognosis of ESCC. Our findings unraveled a high expression of BCAR4 in ESCC tissues and cell lines. Moreover, we found that BCAR4 RNAi suppresses ESCC cell growth and migration via regulating p53/p21 signaling pathway. These results may provide a new rational therapeutic target for ESCC.

## Supplementary Material

Supplemental Material

## Data Availability

All datasets can be requested from the corresponding author.

## Highlights

1. LncRNA BCAR4 expression is elevated in ESCC tissues and predicted poor prognosis.

2. Knockdown of LncRNA BCAR4 accelerated cell apoptosis, while suppressed cell proliferation and migration of ESCC cells.

3. LncRNA BCAR4 sponged miR-139-3p to upregulate ELAVL1 in ESCC cells.

4. LncRNA BCAR4/miR-139-3p/ELAVL1 promotes apoptosis in ESCC cells by modulating p53/p21 signaling pathway.

## Abbreviations

BCAR4, breast cancer antiestrogen resistance 4; EC, Esophageal carcinoma; ESCC, esophageal squamous cell cancer; lncRNAs, Long non-coding RNAs; BC, breast cancer; CC, colorectal cancer; NSCLC, non-small cell lung cancer; miRs, MicroRNAs; ceRNA, competing endogenous RNAs; ELAVL1, ELAV Like RNA Binding Protein 1; 3-’UTR, 3ʹ-untranslated region; cDNA, complementary DNA; SDS-PAGE, sodium dodecyl sulfate polyacrylamide gel electrophoresis; ECL, enhanced chemiluminescence; PI, propidium iodide; TCGA, The Cancer Genome Atlas; IGFBP, insulin-like growth factor binding proteins.
