# Supplementary material for: Long non-coding RNA BCAR4 aggravated proliferation and migration in esophageal squamous cell carcinoma by negatively regulating p53/p21 signaling pathway
Source: Bioengineered. 2021 Feb 19;12(1):682–96. doi: 10.1080/21655979.2021.1887645 (PMC8291806; doi:10.1080/21655979.2021.1887645)
Supplement: Supplemental Material [file KBIE_A_1887645_SM9239.zip › Table S1.docx]

**Table S1. The detailed clinical information of 32 cases of patients with esophageal carcinoma.**

| Features | No. of patients | Percentage (%) |
| --- | --- | --- |
| Age (years) |  |  |
| ≤60 | 18 | 56.3 |
| ＞60 | 14 | 43.7 |
| Gender |  |  |
| Male | 19 | 59.4 |
| Female | 13 | 40.6 |
| T [Infiltrate](D:/360%E5%AE%89%E5%85%A8%E6%B5%8F%E8%A7%88%E5%99%A8%E4%B8%8B%E8%BD%BD/Dict/8.4.0.0/resultui/html/index.html#/javascript:;) |  |  |
| T1 | 4 | 12.5 |
| T2 | 11 | 34.4 |
| T3 | 17 | 53.1 |
| stage |  |  |
| 1-2 | 18 | 56.3 |
| 3-4 | 13 | 40.6 |
| BMI |  |  |
| ≤24.3 | 20 | 62.5 |
| ＞24.3 | 9 | 28.1 |
| Weight |  |  |
| ＜72kg | 16 | 50 |
| ≥69kg | 16 | 50 |
