# Supplementary material for: Long non-coding RNA BCAR4 aggravated proliferation and migration in esophageal squamous cell carcinoma by negatively regulating p53/p21 signaling pathway
Source: Bioengineered. 2021 Feb 19;12(1):682–96. doi: 10.1080/21655979.2021.1887645 (PMC8291806; doi:10.1080/21655979.2021.1887645)
Supplement: Supplemental Material [file KBIE_A_1887645_SM9239.zip › Table S2.docx]

Table S2. the shRNA sequences used in cell transfection.

| Name | Sequence (5′→3′) | Concentration(ng/µL) |
| --- | --- | --- |
| shBCAR4-1 | GGGACTTGAGTTATGTTGGTGGCTA | 650 |
| shBCAR4-2 | AAATGAAATGGTCAACTGAAA | 650 |
| shBCAR4-3 | CTGGATTAACAATGATAGCCA | 650 |
| sh-Control | GTTCTCCGAACGTGTCACGTT | 650 |
