# Supplementary material for: Long non-coding RNA BCAR4 aggravated proliferation and migration in esophageal squamous cell carcinoma by negatively regulating p53/p21 signaling pathway
Source: Bioengineered. 2021 Feb 19;12(1):682–96. doi: 10.1080/21655979.2021.1887645 (PMC8291806; doi:10.1080/21655979.2021.1887645)
Supplement: Supplemental Material [file KBIE_A_1887645_SM9239.zip › Table S3.docx]

Table S3 Primers used for qPCR

| Primer name | Sequence (5′→3′) |
| --- | --- |
| BCAR4 Primer-F | AGAAGTCGTCCTGTCGTCCT |
| BCAR4 Primer-R | AGGAGAGACTGGGGGCAAAT |
| ELAVL1 Primer-F | ATGAAGACCACATGGCCGAAGACT |
| ELAVL1 Primer-R | GTTCACAAAGCCATAGCCCAAGC |
| miR-139-3p Primer-F | AAGCCCTTACCCCAAAAAGTAT |
| miR-139-3p Primer-R | CTTTTTGCGGTCTGGGCTTGC |
| U6 Primer-F | CTCGCTTCG GCAGCACA |
| U6 Primer-R | AACGCTTCACGAAT TTGCGT |
| p53 Primer-F | TCTGACTGTACCACCATCCACTA |
| p53 Primer-R | CAAACACGCACCTCAAAGC |
| p21 Primer-F | TGTCCGTCAGAACCCATGC |
| p21 Primer-R | AAAGTCGAAGTTCCATCGCTC |
| GAPDH Primer-F | TGACTTCAACAGCGACACCCA |
| GAPDH Primer-R | CACCCTGTTGCTGTAGCCAAA |
