# Supplementary material for: Long non-coding RNA BCAR4 aggravated proliferation and migration in esophageal squamous cell carcinoma by negatively regulating p53/p21 signaling pathway
Source: Bioengineered. 2021 Feb 19;12(1):682–96. doi: 10.1080/21655979.2021.1887645 (PMC8291806; doi:10.1080/21655979.2021.1887645)
Supplement: Supplemental Material [file KBIE_A_1887645_SM9239.zip › Table S4.docx]

Table S4 Antibodies used in western blotting

| Name of antibody | Protein size (kDa) | Diluted multiples | Source of primary antibody | Company | Number |
| --- | --- | --- | --- | --- | --- |
| p53 (WB) | 53 | 1:1000 | Rabbit | Proteintech | 21891-1-AP |
| p21(WB) | 21 | 1:1000 | Rabbit | Cell Signaling Technology | #2947 |
| HRP Goat Anti-Rabbit IgG | _ | 1:3000 | _ | Beyotime | A0208 |
| β-Actin (WB) | 42 | 1:1000 | Rabbit | Cell Signaling Technology | #4967 |
