# Supplementary figures and images for: Long non-coding RNA BCAR4 aggravated proliferation and migration in esophageal squamous cell carcinoma by negatively regulating p53/p21 signaling pathway
Source: Bioengineered. 2021 Feb 19;12(1):682–96. doi: 10.1080/21655979.2021.1887645 (PMC8291806; doi:10.1080/21655979.2021.1887645)

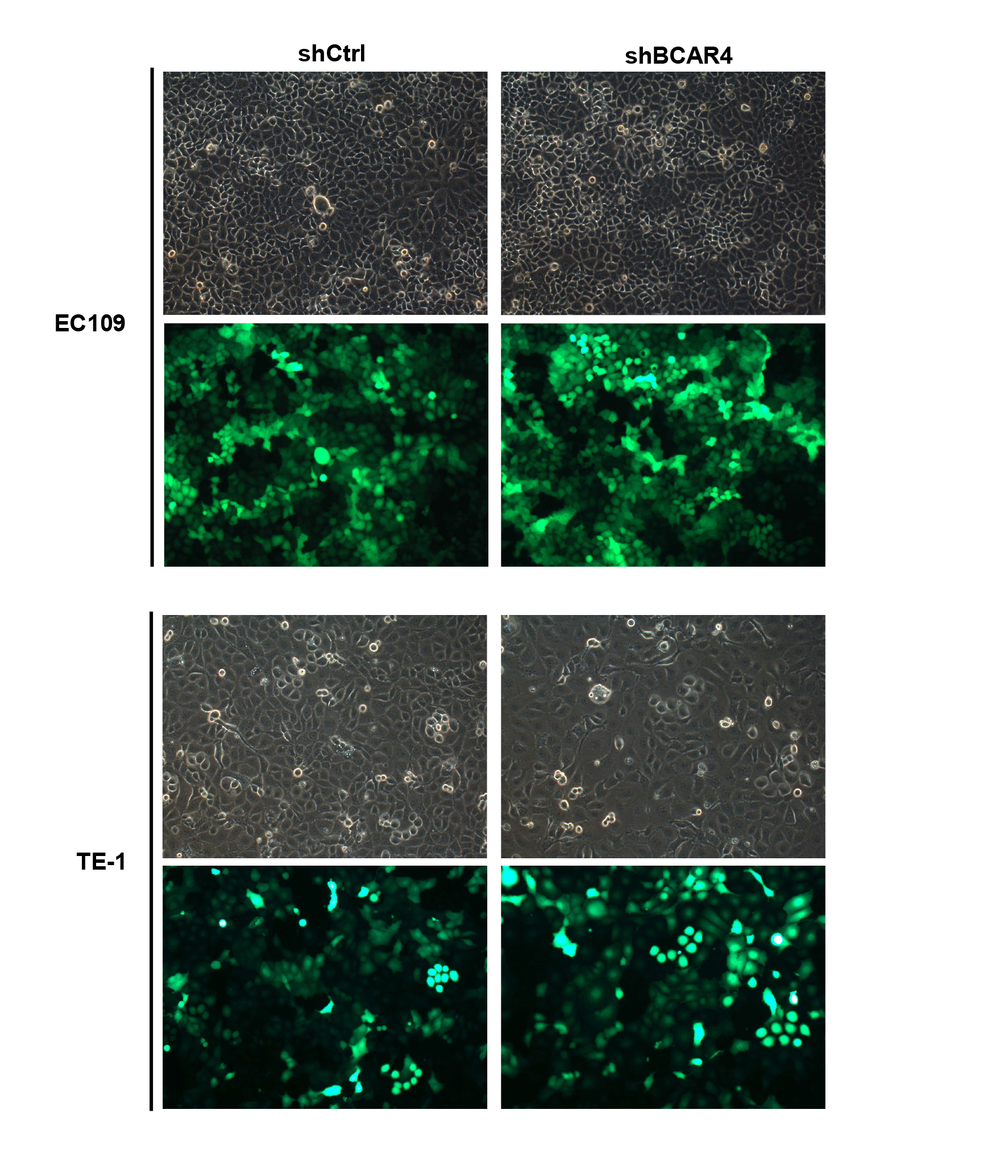

Supplement: Supplemental Material [file KBIE_A_1887645_SM9239.zip › Figure S1.png]
